# To what extent does recurrent government health expenditure in Uganda reflect its policy priorities?

**DOI:** 10.1186/1478-7547-8-19

**Published:** 2010-10-20

**Authors:** Frederick Mugisha, Juliet Nabyonga-Orem

**Affiliations:** 1 Economic Policy Research Centre, Makerere University Kampala; 2 Health Systems Unit, World Health Organization, Uganda Country Office

## Abstract

**Background:**

The National Health Policy 2000 - 2009 and Health sector strategic plans I & II emphasized that Primary Health Care (PHC) would be the main strategy for national development and would be operationalized through provision of the minimum health care package. Commitment was to spend an increasing proportion of the health budget for the provision of the basic minimum package of health services which was interpreted to mean increasing spending at health centre level. This analysis was undertaken to gain a better understanding of changes in the way recurrent funding is allocated in the health sector in Uganda and to what extent it has been in line with agreed policy priorities.

**Methods:**

Government recurrent wage and non-wage expenditures - based on annual releases by the Uganda Ministry of Finance, Planning and Economic Development were compiled for the period 1997/1998 to financial year 2007/2008. Additional data was obtained from a series of Ministry of Health annual health sector reports as well as other reports. Data was verified by key government officials in Ministry of Finance, Planning and Economic Development and Ministry of Health. Analysis of expenditures was done at sector level, by the different levels in the health care system and the different levels of care.

**Results:**

There was a pronounced increase in the amount of funds released for recurrent expenditure over the review period fueled mainly by increases in the wage component. PHC services showed the greatest increase, increasing more than 70 times in ten years. At hospital level, expenditures remained fairly constant for the last 10 years with a slight reduction in the wage component.

**Conclusion:**

The policy aspiration of increasing spending on PHC was attained but key aspects that would facilitate its realization were not addressed. At any given level of funding for the health sector, there is need to work out an optimal balance in investment in the different inputs to ensure efficiency in health spending. Equally important is the balance in investment between hospitals and health centers. There is a need to look comprehensively at what it takes to provide PHC services and invest accordingly.

## Background

The National Health policy for Uganda (NHP) 2000 - 2009 [1] emphasized that Primary Health Care (PHC) would be the main philosophy and strategy for national development and would be made operational through provision of the minimum health care package. A minimum health care package, with interventions addressing the biggest burden of diseases affecting majority of the population, would form the primary focus of the health care delivery system. The welfare of the poor was to be given special consideration. Even though there was a reduction in poverty levels - from 38% in 2002/03 to 31% in 2005/2006 -, the population in poverty was still considered significant and more pronounced in rural areas [2,3].

The NHP[1] committed to allocating and spending an increasing proportion of the health budget for the provision of the basic minimum package of health services. This was interpreted during implementation to mean increasing spending at health centre level, where majority of the population, especially the rural and poor, lived and sought public health services (district health services). It is noteworthy that health centers' main focus is primary health care while hospitals' main focus is tertiary care. However, the policy provides for hospitals to offer primary health services in the absence of health centers. Spending at the central level and on referral and tertiary hospitals was to be held constant in real terms. The Health sector strategic plan (HSSP) I; 2000/01 - 2004/05 similarly stated ensuring effectiveness, efficiency, and equity in allocation and utilization of resources and expenditure on most relevant and cost effective priority health interventions, with a clear bias on protecting the poor and most vulnerable [4]. Review of the HSSP I noted that some progress had been made. There was an increase in funding to PHC services in absolute and relative terms targeted at peripheral health units [5].

HSSP II 2005/06 - 2009/10 committed to allocating resources in the health sector in line with efficiency and equity principles[6]. Preferential allocation of resources to cost effective activities and increasing consumption of services were measures envisaged to address allocative and technical efficiency issues respectively. Targeting increasing allocations to health care inputs with a large impact on quality of services especially drugs and other health supplies, and increasing the proportion of resources allocated to the district health services where the majority of population, especially the poor, live were among the strategies to address equity concerns.

Uganda has a decentralized system of health service delivery with roles and responsibilities for the centre and districts clearly stated. The central level is responsible for setting policies, standards and guidelines, resource mobilization, capacity building, coordination of service delivery, monitoring and supervision. The decentralized levels (districts) are responsible for service delivery.

The objective of this paper is to provide a better understanding of changes in the way recurrent funding (wage and non-wage) is allocated in the health sector in Uganda and to what extent it has been in line with agreed policy priorities.

Understanding changes in the way funding is allocated provides government and development partners an opportunity to examine not only the importance attached to its priorities but also because it provides a tool for monitoring the benefits expected from increased spending. In general expenditure on health is one of the determinants of health status. For example, in Lesotho, public expenditure on health was one of the important determinants of life expectancy at birth, infant mortality and under-five mortality [7]. Similar results were found in Pacific Island Countries while examining the relationship between per capita public expenditure and three measures of health outcomes (infant and under-five mortality rates and crude death rates)[8]. The results showed strong evidence that per capita health expenditure is an important factor in determining health outcomes. More important is the fact that when this expenditure is targeted, it is expected to yield better outcomes than when it is not. For example, Vietnam introduced a Health Care Fund for the Poor to increase access to health care and reduce the financial burden of health expenditure faced by the poor and ethnic minorities. The results suggest that despite numerous administrative challenges, the fund helped increase utilization and reduced out-of-pocket expenditure for the program's target population[9].

## Methods

### Data

Quantitative research methods were used. The main dataset used in this paper is the government recurrent wage and non-wage expenditures - based on outturns by the Uganda Ministry of Finance, Planning and Economic Development (MoFPED). Expenditure outturns are actual releases of funds from central government to various spending centers. Possible alternative expenditure data is that based on actual accountability for expenses made at the end of each financial year. This data is unavailable. Accountability for expenditure is meant for audit purposes and therefore is not consistently captured.

The expenditure data based on central government outturns was compiled from approved estimates of revenue and expenditure reports that MoFPED publishes annually [10]. The data was complied for ten financial years - from the financial year 1997/1998 to financial year 2007/2008. This data does not include funds that are recurrent in nature not expended through the common government basket. Additional data was obtained from a series of annual health sector reports [11-13] as well as other reports.

Data was verified by key government officials in MoFPED and Ministry of Health (MoH). The key aspect required in the verification exercise was to confirm whether the data was correct and provide input in explaining the observed patterns. The Ministry of Finance, Planning and Economic Development being the source of the data provided all the necessary data reports. Officials from the planning department of the Ministry of Health responsible for finance and budget - given the subject matter - were asked to review the data and also comment on the emerging trends. The authors shared with them the data and the paper. They made few collections on the figures and shared with the authors their own compilation - which were reconciled.

### Analysis

In order to understand the pattern of and further examine the recurrent government expenditure for PHC, the recurrent budget was categorized into two service care levels - the hospital services and the health center services. The Uganda health service structure is organized broadly in this manner. The primary health care services include services offered at Health Center I or Village Health team, Health Center II, Health Center III, and Health Center IV. Hospital services are provided at a General Hospital, Regional Referral Hospital and the National Referral Hospital.

**Figure 1:**
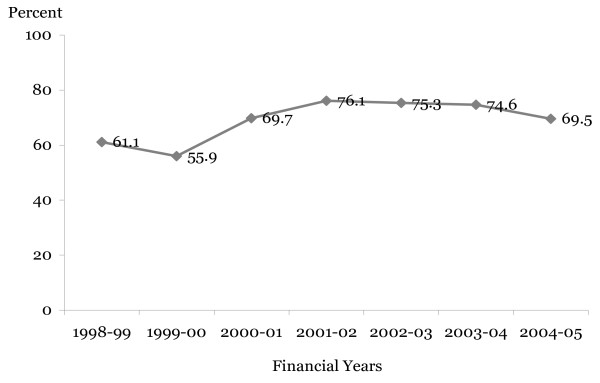
**Percent government NGO funds spent on hospital services**. Source: Medical Bureaus databases; annual health sector reports

The structure of the health sector outturns as presented in the estimates of revenue and expenditure is shown in Table 1. Hospital expenditure includes that of Mulago Hospital Complex, Butabika Hospital, Regional Referral Hospitals, General Hospitals and NGO hospitals. NGO subvention is spent at NGO Hospital and Health Centre levels. The percent of NGO expenditure on hospitals was used in computing the actual expenses for hospital services. The part of the district NGO hospitals or primary health care that is spent on hospital care was varied across the years (see Figure 1).

**Table 1:** Example of the Health Sector Budget structure

FY 2007/08 Out-turn					
**SECTOR/VOTE HEALTH**		**Wage**	**Non-Wage Recurrent**	**Domestic Dev**	**Donor Project**

14	Health	2.86	42.78	19.62	
107	Uganda Aids Commission (Statutory)	0.69	0.63	1.51	
134	Health Service Commission	0.45	1.25	0.04	
151	Uganda Blood Transfusion Service (UBTS)	0.84	0.85		
161	Mulago Hospital Complex	12.42	17.16	1.32	
162	Butabika Hospital	1.58	1.8	6.8	
163-173	Regional Referral Hospitals	16.93	8.82		
501-850	NGO subvention		15.93		
501-850	District Primary Health Care	78.9	19.85	6.31	
501-850	General Hospitals		9.61		

	SUB-TOTAL HEALTH	114.66	118.7	35.6	

Source: MFPED, Estimates of revenue and expenditure (recurrent and development) FY 2007/08. 2008, Ministry of Finance, Planning and Economic Development: Kampala, Uganda.

The total expenditure on PHC is shown as "District Primary Health Care" plus the amount expended on NGO Health Centers. Expenditure by the MoH headquarters and district health offices is included as a separate category. The MoH headquarters and district health offices do conduct management functions on behalf of the Health Centers and Hospitals.

Expenditure on Uganda Aids Commission, Health Service Commission and Uganda Blood Transfusion Service is considered to be other expenditure. Other expenditure items that have been phased out over the years include the Health Training Schools and the lunch allowances. The training schools were transferred to be under the Ministry of Education while the lunch allowance was consolidated in the wage component.

## Results

### Overall Recurrent Expenditure

**Figure 2:**
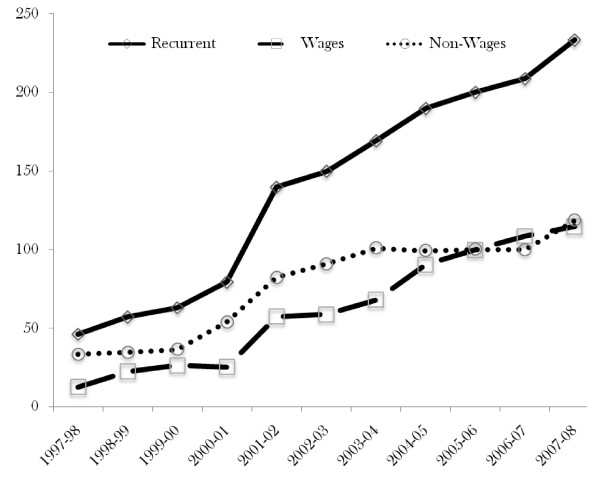
**Recurrent expenditure for the health sector in Uganda - Ushs (billions)**.

Figure 2 shows recurrent (wage and non-wage) expenditure for the health sector over a period of ten financial years.

Three messages are noticeable from the results. First, during the year 2000-01, there was a pronounced increase in the amount of funds released for recurrent expenditure. It increased by about UGX 60 Billion or 76 percent between 2000/01 - 2001/02. This coincided with Government of Uganda policy to focus on PHC as a strategy.

Second, prior to 2005-06 the non-wage component of recurrent expenditure was higher than the wage. However after this financial year, the wage component caught-up with the non-wage component.

Third, we notice that the wage component of the recurrent expenditure increased consistently each year during the period under review while the non-wage did not show significant increase. This suggests that increases in the wage component contributed most to the increase in recurrent expenditure.

### Recurrent Expenditure Across Levels

**Figure 3:**
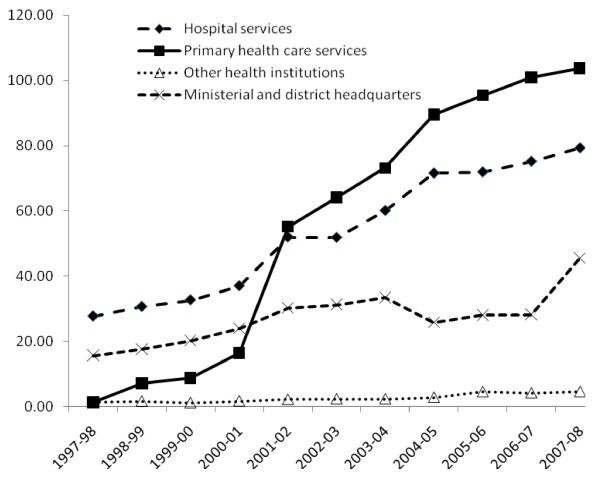
**Categories of recurrent expenditure in the health sector (Billion UGX)**.

Figure 3 shows the recurrent expenditure across different levels in the health system.

PHC services showed the greatest increase over the ten year period. Between 1997 and 2000, primary health care services attracted less recurrent expenditure than hospital services or the ministerial and district headquarters. In the financial year 2000-01, a policy directive was made to focus spending on PHC as a strategy to bring services closer to the population. Indeed, following this pronouncement, in the financial year 2001-02, expenditure on PHC shot up beyond that of hospital services. The rate of increase after this time continued to be higher than that of hospital services. Recurrent expenditure at district and ministry headquarters, and other health institutions remained rather constant over this period. In the rest of the paper, no further analysis of ministerial and district headquarters is done. Further analysis was done for hospital services and primary health care services.

### Recurrent Expenditure On Phc Services

**Figure 4:**
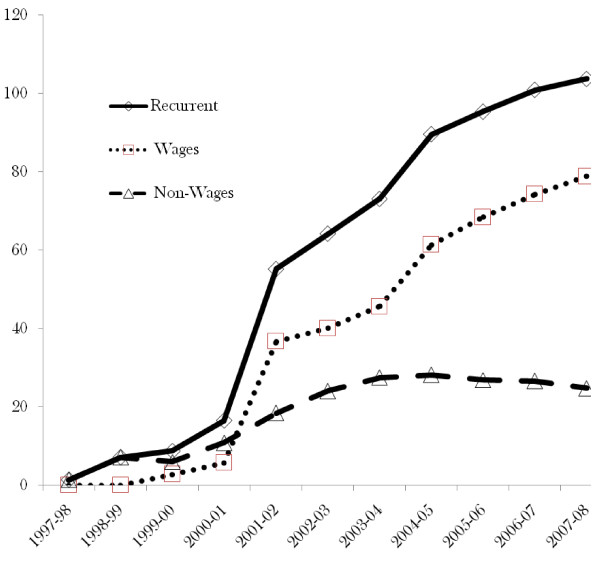
**Primary health care recurrent expenditure - Uganda shillings (billions)**.

In line with the policy decision, recurrent expenditure on PHC services has gone up from 1.5 billion in 1997-98 to 106 billion in 2007-08 in nominal terms, increasing more than 70 times in ten years (see Figure 4). We notice that the increase was mainly driven by wage component of the recurrent expenditure. In fact the non-wage component actually started to decline in 2003/2004 and continued to decline.

**Figure 5:**
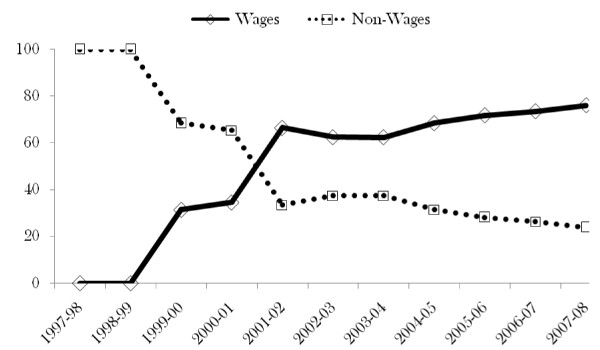
**Primary health care recurrent expenditure - percent**.

Considering the wage and non-wage components as percentages of the total, Figure 5 shows that the percentage of non-wage has continued to decline. This is in contrast to the wage, which moved from a negligible percentage to about 80 percent in 2007-08. This result creates an impression that expansion of health services was pushed by the wage component with increasingly less resources to deliver the services.

### Recurrent Expenditure On Hospital Services

**Figure 6:**
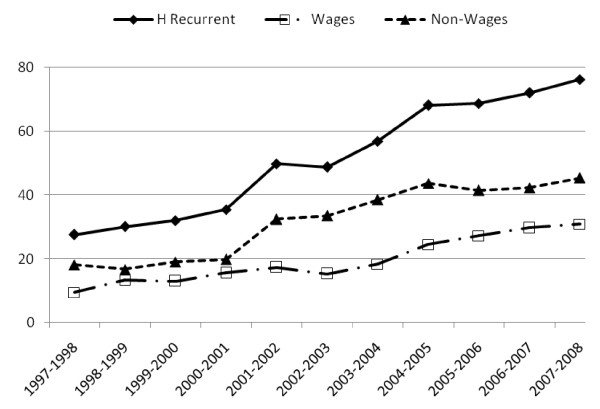
**Hospital level recurrent expenditure - Uganda shillings (billions)**.

Recurrent expenditure for hospital services went up from 27 billion in 1997/1997 to 76 billion in 2007/2008 in nominal terms, increasing almost 3 times in ten years (Figure 6). Unlike with PHC services, non-wage expenditure was consistently higher than the wage component. The expenditure pattern at hospital level differs from that seen at PHC level.

**Figure 7:**
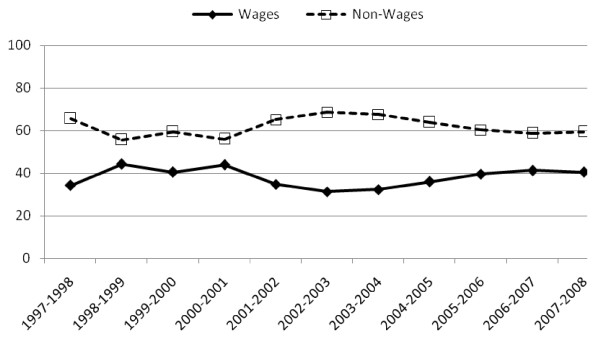
**Hospital level recurrent expenditure - percent**.

Figure 7 shows the two components of recurrent expenditure - wage and non-wage - in terms of percentage. The results show that, unlike the expenditure on PHC services that on hospital services is consistent. The percent expenditure on wage is consistently lower than that of non-wage for all the years.

## Discussion

We begin the discussion with a comment on the scope of the paper. The paper does examine recurrent government recurrent expenditure. It therefore leaves out government development expenditure. It also leaves out expenditure that is not done through the government medium expenditure framework (MTEF), that is, the off budget expenditure. This off budget expenditure is done mainly by development partners and non-government organizations. The proportion of the government recurrent expenditure to the total expenditure of the sector is difficult to estimate. No data is collected either for the non-government organizations or for the development partners. However, analysis of recurrent expenditure in itself is a an important aspect of understanding government priorities.

Simultaneous changes as part of the implementation of the policy are noteworthy. A one off capital investment for Primary Health Care was made four years into the implementation of the policy. Capital expenditure increased from Ushs 10.6 billion in the 2001-2002 financial year to Ushs 75.8 billion shillings in the next financial year (2002-2003) - Based on analysis of the mid-term expenditure framework. This investment enabled further expansion of health centers with an additional 400 HCIIs. In addition, the recruitment of health workers was re-instituted in 2001. By mid 2002, more than 85% (2,538) of health workers under the Poverty action funds targeting PHC services had been recruited[5].

The sector wide analysis has shown an increase in recurrent expenditure largely accounted for by increase in the wage component while the non wage recurrent remained fairly constant. The noted increase in the wage bill may be explained by several reasons, increase in salaries, adjustment for inflation and recruitment of more health workers. We note that increase in public servants wages have been very modest, for example, between 1999 and 2002 wages grew by 4.8% per annum[14] while between 2002 - 2007, average monthly earnings only increased by 13%[15]. Inflation was maintained below 8% between 2000 and 2008[16]. Percentage of approved posts filled by trained staff in the health sector improved from 33% in 1999/00 to 69% in 2004/05[11] and given this, increase in number of health workers accounts for the increasing wage bill.

The availability of human resource without adequate inputs affects quality of care and leads to further loses, having to pay salaries for health workers who are not providing services. Underfunding other key inputs has affected service delivery, for example, medicines stock outs is a long standing problem. The percentage of health facilities registering stock outs in essential medicines has consistently been over 60% for the last 10 years [11-13]. Per capita expenditure on essential medicines and health supplies has remained below US$2 per capita for the last six years [11-13,17-19]. Functionality of available equipment ranges from as low as 33% at the general hospital level to 52% at the Health Centre four (HC IV) level[19]. Available investments cannot be put to optimal use.

Looking at recurrent expenditure by level, the policy decision of capping of expenditures at tertiary level hospitals, which were viewed to serve a small urban population was achieved. Similarly, the high and increasing recurrent expenditure at the district level shows that the policy decision to target peripheral units serving mainly the poor [17]was also met. Eighty percent (80%) of the population live in rural areas and poverty is more prevalent in rural areas compared to urban areas However, expenditure at the national level health institutions remained the lowest and constant over the review period. These institutions offer support services and their proper functioning is crucial for realization of PHC provision. As a result of underinvestment in these institutions, key aspects of PHC have not been effectively provided. Inadequate blood transfusion services for example have affected delivery of emergency obstetric care services. The mid-term review report noted the markedly inadequate and poorly furnished blood transfusion structure and lack of enough blood supply[18]. Out of the seven recommended regional blood banks, only five are in place with no expansion made for the last three years[20]. Operations of the Health service commission, charged with the responsibility of establishing an efficient health workforce continue to be constrained[18]. Occasionally, funding for the wage bill has been returned to the treasury because of failure to recruit health workers.

The patterns of expenditure within hospitals and health centers have been analyzed. We have noted that between 2000/01 and 2001/02, at the hospital level, there was a reduction in the wage and an increase in the non wage recurrent while at the health centre level, there was a reduction in the non wage recurrent and an increase in the wage. This has affected availability of essential supplies, for example, percentage of health centre IIs experiencing stock outs of essential medicines has been close to 80% for the last 3 years compared to the national average of close 70%. Only 52% of HC II were able to provide antenatal care compared to hospitals at 95% [21]. Percentage of HCIIs, offering child immunization with all equipment available, was only 55% compared to hospitals at 90%. Preferential investment in wage at the expense of the non wage recurrent at HC level has affected service delivery at this level with resultant congestion at hospitals of cases that could be treated at lower levels. Similarly, evidence suggests that human resource utilization is suboptimal when investment in other areas like of the capital (i.e. medical equipment, etc.) is inadequate. They recommended that reorientation of the resource allocation towards the capital investments would save more lives [22].

## Conclusion

In this analysis we have shown that the policy aspiration of increasing spending on PHC was attained but key aspects that would facilitate its realization were not addressed. Support services like blood transfusion and the human resource commission which facilitates recruitment of health workers were not supported. At any given level of funding for the health sector, there is need to work out an optimal balance in investment in the different inputs to ensure efficiency in health spending. Much as improving availability of health workers is important, they must have necessary inputs to provide services. Equally important is the balance in investment between hospitals and health centres. There is need to look comprehensively at what it takes to provide PHC services and invest accordingly.

## Competing interests

The authors declare that they have no competing interests.

## Authors' contributions

Both authors contributed substantially in the conception and design of the paper, acquisition of data, analysis and interpretation of data. Both authors drafted the manuscript, revised it critically for important intellectual content and gave final approval of the version to be published.
